# Report on Cholera in Chicago in 1866

**Published:** 1867-07

**Authors:** W. R. Marsh

**Affiliations:** Chicago


### CHICAGO MEDICAL JOURNAL.

VOL. XXIV. JULY, 1867. No. 7.

### Original Contributions.

### REPORT ON CHOLERA IN CHICAGO IN 1866.

CHICAGO, May 20th, 1867.

PROF. J. ADAMS ALLEN,

*Ch'n Com. on Prac. Medicine and Epidemics, Ill. State Med. Society:*

SIR:—In compliance with your request, I submit the following Report on Cholera in Chicago in 1866, as made up from observation, from conference with other medical gentlemen, and from the records of the Health-Office.

In regard to other epidemics, none have prevailed to any great extent, and I have gathered no facts of sufficient interest to bear detailing.

Very Respectfully,

W. R. MARSH.

*The Cholera*, the only disease prevalent to any extent in Chicago, during the past year, was first reported at the Health-Office as having made its appearance at No. 282 West Chicago Avenue July 21st, 1866. This case, as the record states, "being the first case reported for 1866, was not published." The record continues, "the patient died," which, however, is an error, as Dr. R. S. Addison, who reported the case, assures me the case recovered.

Prof. N. S. Davis reported to the Chicago Medical Society that, as early as in June, he had treated three cases of diarrhœa, in which the patients had rice-water discharges, and that, during that month, there had been a marked tendency to increase of bowel diseases with "cramps."

This latter fact was noticed by other physicians, and in several instances they remarked that, were the cholera prevailing in the city, they should not hesitate to pronounce the disease for which they were treating certain of their patients to be cholera. This careful avoidance of the term cholera, induced, perhaps, by a lurking fear that they might be considered alarmists, continued until August 14th, following, when three fatal cases, two in the North, and one in the South Divisions of the city were reported. From this date, more or less cases were reported daily, up to November 3d. A few cases were reported on the 8th, and the next and last on the 23d of November.

The record at the Health-Office shows that, during this time, reports were received of 1581 cases. The record of the Health-Office gives a weekly and monthly exhibit of all the cases reported, from the first case, on the 21st July, to the final disappearance of the malady, November 24th:—

| | | | |
|---|---|---|---|
| 1st Week, | 1 | 9th Week, | 74 |
| 2d " | 0 | 10th " | 57 |
| 3d " | 0 | 11th " | 63 |
| 4th " | 37 | 12th " | 532 |
| 5th " | 98 | 13th " | 397 |
| 6th " | 80 | 14th " | 83 |
| 7th " | 65 | 15th " | 24 |
| 8th " | 61 | 16th " | 7 |
| On the 23d November, | | | 2 |
| Total No. of cases, | | | 1581 |

| | |
|---|---|
| During the month of July, | 1 |
| " " " August, | 215 |
| " " " September, | 268 |
| " " " October, | 1082 |
| " " " November, | 15 |
| Total, | 1581 |

The largest number reported on any one day was 175, on the 10th October; the second largest, 108, on the 11th October, from which date the epidemic irregularly declined up to October 31st, when only one case occurred. Of the number attacked, 970 died. The census taken by the school authorities, gives the population of the city as 200,330. This being correct, the mortailty from cholera was 1 in 206 of the inhabitants, and the number of those attacked was 1 in 126, fractions in either case not being considered. These figures show a smaller per cent of attacks and fatal results to the number of population than the average in cities of this or foreign countries in former epidemics, but the mortality among those attacked is 61.539 per cent, which is fully equal, and a little above, the average.

But the question arises, to what extent are the records of the Health-Office reliable? I answer, that they are as perfectly so, as to the cases reported and their results, as any such records are, but they are only an approach towards an exhibit of the facts. This imperfection arises from the defect in our registry laws regarding deaths and burials. The physician's certificate is not required, and the cause of death is always, or nearly always, recorded as given by the friends or other parties, who are of course incompetent, in a very large proportion of cases, to assign the real cause, and, oftentimes, of giving anything like a near approach to the truth.

The result of this is plainly to be seen in the Health-Officer's reports of causes of death each month and year, where such causes as the following are constantly appearing:—"fever," "disease of the bowels," "fits," "cramps," etc., etc.

In the last annual report of the Chicago Health-Officer, 112 deaths are attributed to "cramps," nearly, or quite, all of which should, doubtless, be ascribed to cholera, thus swelling the number of deaths from this source to 1082; and if we suppose all attacks to have been reported, this would increase the mortality from about 61½ to over 68 per cent.

This conclusion we cannot for one moment admit, for it is too well known that many cases of undoubted cholera were not reported by attending physicians, while the deaths, in a number of instances, occurred where no medical attendance was had, and, consequently were included in the 970 deaths, but not in the list of 1591 of those attacked. In my own practice, more cases by far of choleraic diarrhœa, or "cholerine," were treated than of patients reported as the cholera, and I am assured by several physicians of extended practice, that they had like experience. These cases mostly yielded readily to treatment, and where the more serious choleraic symptoms did not follow, no public record was made of them. Such a course was erroneous, since it renders the statistics altogether incomplete, but it was the custom, or at the least it saved the physician more or less trouble, and was, I think, very generally practiced. One fact, in evidence of this, a journal contains an article from a physician in which he claims to have cured, to say nothing of fatal cases, quite a number more of patients than the Health-Office shows him to have reported.

In short, from my own observation and from conversations with intelligent medical men upon the subject, I am convinced that to add 1000 to the number attacked, as reported by the record, making 2581 instead of 1581, and to add the 112, as above, to the 970 deaths recorded, making 1082 deaths, would be a much nearer approximation to the truth than are the statistics above given.

If such hypothesis be correct, we have, one person attacked in every seventy-seven and a fraction, one death in every one hundred and eighty-five and a fraction of the population, and the mortality 41.92 per cent of those diseased. In either case, it appears that the epidemic was a lamentable catastrophe to the city, and supposing the cases to have been treated by medical attendants, the results were anything but flattering in the way of success. Mortifying as it is to our self-love and to our anticipations of what we, as a profession, should find our ability to control the disease to be, as compared to our former experience and that of our predecessors, we are forced to concede the fact, that our progress may be, very nearly, represented by a zero.

And, truth to say, I am unable to perceive, from a somewhat careful perusal of the literature of this disease, that any very marked changes in the mode of treatment have been proposed, or attempted with unusual success, in this epidemic, either here or elsewhere. Nor, in the pathology of the disease, do I see that the profession have made undoubted progress, of great magnitude, even since the epidemic of 1831–2. Much discussion has been had upon both questions, and many changes rung on words, but all with very slight modification of ideas.

A few, indeed, I may say, many new remedies have been proposed as theoretically valuable, but, for the most part, they have failed in the trial, or proved in no wise more valuable than those before employed, and our best men, everywhere, have, in the main, found themselves more successful in relying upon the experience of the past than in their experiments with untried measures proposed.

Pathological researches have been made, too, in abundance, and particular facts developed, but, as a whole, I do not see that any material advance has been made upon the combined theories of Binaghi and Dupuytren, in 1831, the one of whom says, "it is a disease of the *nervous system*, and particularly of the *nervo-splanchnic* system," and the other, that "it has its seat in the alimentary canal, and consists of an irritation of the secretory apparatus, and especially of the small glands or follicles of the intestines." "Blood changes," diminution of vital affinity," may come before; or "removal of the columnar epithelium of the villi of the intestinal mucous membrane" come after, but that both these observers are correct, and that the phenomena of the distemper are chiefly referable to the systems of which they speak, is incontrovertible thus far.

Without stopping, however, to discuss this question, which would be out of place since I propose to offer nothing new, we will turn to a more cheerful topic, namely, the *prevention* of cholera. If we have made little progress in the cure here, a world of good has been gained, for we have learned that proper sanitary and hygienic measures are competent to deprive the cholera of its terrors almost as certainly as has vaccination those of small-pox.

This fact has been demonstrated clearly in statistics of American and foreign cities and towns. In Chicago it is apparent also, as in other cities, but save to those who know well the city, its general typography and the exact extent of its improvements, it is not easy of demonstration. In an examination of the subject it is necessary to bear in mind that ours has been, in some respects, justly regarded on the whole, as a *dirty* city. But a few years since it was variously denominated a "swamp," a "bog," a "mud-hole," and other names, all signifying that it is situated upon land, level, and so little elevated above the river and Lake Michigan, as to be continually wet, thus being a wet prairie or marsh. Of that portion which public enterprise has not redeemed by draining and raising the surface by filling with earth, much of the land is still of that character, some portions even of that being occupied by dwellings, being subject to overflow from the river at each period of high water.

In but little more than the time of half a generation, it has grown from a little village until it has come to contain over two hundred thousand souls, and its borders have spread from little more than what comprises two of the smallest of its present sixteen wards, to cover a space measured by miles in each direction. Its 83½ miles of public sewerage, its water-works, its many raised and paved streets, and its public buildings, its churches and schools, all testify to its remarkable public enterprise. But where was there ever a country, a city, or, I had nearly asked, even the individual who prized the preservation of health above the acquisition of wealth, or would not risk the former, if need be, to procure the latter? Sanitary improvements have always followed, not kept pace, with other improvements whether public or private. The sewers of Paris and London are of very modern origin. The pioneer always lives in a cabin until he has built his barns. And so while Chicago, considering her age, has accomplished more in the way of sewerage and in completing an artificial elevation of her streets and general surface than is recorded of any city of her "age and size" it still remains true, to quote an article in a recent daily paper, the *Tribune*, "that by far the largest part of the streets within the city limits has no drainage except that from the slight slope produced by nature. We have about 600 miles of streets, of which a little less than one-seventh are drained at all. Many of the lots passed by these sewers are not connected with them by drains, so that a large proportion of our surface loses its surplus moisture by the time-honored way of evaporation. Probably about one-ninth of the land passed by sewers, is not connected with them by sub-drains. The greatest part of the sewage is carried into the river, the heavy portion sinking to the bottom, forming a sediment which is stirred up by the movement of vessels, and the lighter particles floating on the surface until evaporated and returned into the atmosphere. In not very remote times, (indeed until the spring of 1867,) a large portion of this delectable fluid passed into the lake, and was forced by the wind into the basin of the water-works, whence it was returned through the pipes and served up for washing, drinking, and cooking purposes. We should not cease to congratulate ourselves that we now obtain water from the pure depths of the lake, so that the most serious evil of the drainage system is overcome."

To the above evils growing out of our natural surface, we we must add that while many of the streets have been raised to the established grade, this is not true of unoccupied lots generally, and, indeed, of many of the occupied lots, and as a necessary result, where such lots are not connected by drains with the sewers, their condition is tenfold worse than before.

Again large portions of the city were not supplied at all, and others only partially, with water from the public water-works, and, bad as this was hitherto, the inhabitants were driven to even a worse expedient, namely, the use of water from shallow wells contaminated with the percolations from a thousand stables, privies, and the cemeteries of the dead.

These general remarks seem necessary, in order to explain certain facts we are about to give respecting the prevalence of cholera in the different wards of the eity. Without them, these facts would seem to conflict with what are given as facts in other cities and localities. With them, and the few remarks to be made after giving the figures, no such misunderstanding need to arise. Referring then to the report of the Health Office as the only record of the statistics in Chicago, and which if they be as imperfect as we have before supposed, should yet be considered as representing the facts as nearly accurately in one portion of the city as in another, and we find as follows:

In the 1st, 2d, and 16th wards, containing 37,589 inhabitants, or less considerably than one-fifth of the population, nearly or quite three-fourths of the public improvements in sewerage, of graded and paved streets, and quite the half, I should judge of the wealth, and the comforts which wealth brings, of the entire city, furnished nearly one-fourth, 376, of the cholera cases reported. If to these we add travellers and those attacked in hotels and prison, nine-tenths of whom without a doubt were attacked in these wards, we then have 420 cases, or about 25 more than one-fourth of the whole.

In the 7th ward, with a population of 18,754, with no streets improved more than common country highways, and with very little sewerage, and population composed almost exclusively of the poorer classes of foreigners—in this ward with almost half the population of the three first mentioned, we find 179 cases reported.

In the 11th, 12th and 15th wards, with a population of 41,300, with sewerage and streets in a like condition with those of the 7th ward, we find reported 434 cases.

In the other nine wards, with very few improved streets, and as compared with the first three wards enumerated, having but little sewerage, but containing in the aggregate a population of 102,697, there were only 528 cases reported.

At first view of these facts it would seem that improvements, intended to exert a healthful, had, on the contrary, exerted a baneful influence upon the city, but recalling the remarks already made on the situation of the city, and with a few particulars respecting peculiarities of the different wards and such conclusion is readily seen to be false.

Thus the 1st and 16th wards on two sides, and the 2d ward on one side, have the river, the character of whose waters has been described, as a boundary. As a partial offset to this, on the east side of each is the lake.

These wards also comprise but a comparatively small area of land. They make up the business part of the city—contain all the principal hotels, the prisons, public buildings generally and nearly all the business houses. In these all travel centres and all public business calling together large masses of people are held. Notwithstanding that their streets are paved and their sewers built, a large number of their lots are not filled and in the sections where cholera most abounded, crowded tenements on undrained premises, crime and debauchery prevail to an extent unknown in any other part of the city. Outside of these localities and some "patches" along the river banks the disease rarely appeared, and had proper sanitary regulations been strictly enforced I am satisfied would hardly have made its appearance at all.

In all the other wards like facts are found upon careful examination. In examining the sewerage map of the city and the cholera record together, the evidences of the effect of good or bad drainage in the induction of this fell disease are so manifest that our present Sanitary Superintendant has written "cholera" on the map wherever the population is anywise dense and no sewers, and this, because last year's experience demonstrated that in such localities alone did the disease appear in other than what might be termed a sporadic form. In the 7th ward where the largest number of cases are reported as occurring in any one ward of the city, nearly all reported cases occurred in a small district of the ward where, in addition to the want of proper sewerage, the streets were but little, many of them in nowise, better than broad alleys, and the blocks between principal streets intersected in both directions by streets of the same character, and the population of the character before described crowded upon these blocks with their horses and cattle, their pigs and poultry. The same is true of the 6th and 8th wards.

That the 5th ward including Bridgeport and its oft complained nuisances and notorious population, and the 13th with its cemetery associations concerning which there has been so much forboding, should have furnished only 31 cholera cases each, less than any one of the other fourteen, is to be accounted for by the fact of the small number of their inhabitants and the large area of territory which the wards comprise. The prevalence of cholera in these wards was as in all others in proportion to the density of population and the facilities for ridding themselves of accumulations of matters deleterious to health.

I need not recapitulate farther the numerous facts which have come within my knowledge to prove that "cleanliness is next to godliness." Nor do I propose to weary you with arguments to prove the necessity of what, thank heaven, and the in most part unrewarded, and selfsacrificing efforts of our profession has already been demonstrated, namely unless the city become, itself, or find, some Hercules to clean its "Augean stables" we are not yet free from another pestilence of cholera or any other disease.

In closing this report, already too long but made so seemingly if I made any, permit me to ask to what extent was the fatality of cholera in Chicago modified for good or ill by the universal custom among the citizens, of all orders and classes, of keeping by them the "cholera medicines" reccommended generally. I do not know that they did not save many lives, but this I do know, that I saw no fatal case which was not one of extreme collapse, and in which the patient had "doctored" him or herself with some of the popular medicines until too late for me to accomplish anything.

It was at the same time, I think, notoriously true that in the premonitory and early stages of the disease there was comparatively little difficulty, generally, in controling it.

Very Respectfully,

W. R. MARSH.

